# Personality characteristics, alexithymia and anxiety/depression in breast cancer patients undergoing radiotherapy: a cross-sectional mediation analysis

**DOI:** 10.3389/fpsyt.2026.1891800

**Published:** 2026-08-26

**Authors:** Ying Li, Yue Luo, Zheng Li, Dan Wang, Lu Yang, Yan He, Qinglian Wen

**Affiliations:** 1 Department of Radiotherapy, West China Hospital, Sichuan University, Chengdu, China; 2 Department of Cardiology, West China Hospital, Sichuan University, Chengdu, China; 3 West China School of Nursing, Sichuan University, Chengdu, China; 4 Department of Cancer Center, Second Affiliated Hospital, Chongqing Medical University, Chongqing, China

**Keywords:** alexithymia, anxiety, breast cancer, depression, personality characteristics, radiotherapy

## Abstract

**Objective:**

This study aims to explore the relationship between personality characteristics and both anxiety and depression in breast cancer patients undergoing radiotherapy after surgery, and to assess the indirect effects of alexithymia.

**Methods:**

This cross-sectional study was conducted in the Department of Radiation Oncology from January 2024 to May 2024. A convenient sampling method was used to recruit breast cancer patients receiving radiotherapy after surgery. Participants completed the survey during the last two weeks of their treatment. Personality characteristics were assessed using the Eysenck Personality Questionnaire (EPQ), alexithymia using the Toronto Alexithymia Scale (TAS-26), and anxiety/depression using the Hospital Anxiety and Depression Scale (HADS). Correlation and mediation analyses of anxiety, depression, personality characteristics, and alexithymia were conducted using SPSS version 27.0 with the PROCESS macro.

**Results:**

A total of 231 patients were included in the analysis. The rates of probable anxiety and depression among breast cancer patients undergoing radiotherapy after surgery were 34.6% and 51.5%, respectively. Neuroticism and psychoticism were positively correlated with both anxiety and depression (r ranging from 0.246 to 0.571, all *P* <.05), while extraversion was negatively correlated with depression (r = -0.208, *P* <.01). Alexithymia was positively associated with both anxiety and depression (r = 0.294 and 0.407, respectively, both *P* <.001). Mediation analysis revealed that alexithymia showed significant indirect effects in the relationships among personality characteristics, anxiety, and depression (indirect effects: -0.027 to 0.020, all 95% confidence intervals excluded 0).

**Conclusions:**

This study suggests that personality characteristics are associated with anxiety and depression in breast cancer patients undergoing radiotherapy, with alexithymia showing significant indirect effects in these relationships. Given the cross-sectional design, these findings represent indirect associations rather than causal pathways.

## 1 Introduction

Breast cancer is the most common cancer among women, with over 2,261,419 new cases and 684,996 deaths reported worldwide annually (1). Radiotherapy, an essential component of comprehensive breast cancer treatment, plays a crucial role in improving patient survival (2). However, radiotherapy can cause various physical symptoms, including fatigue, pain, dermatitis, and edema, which may reduce patients’ quality of life and contribute to negative emotions such as anxiety and depression (3). The reported rates of anxiety and depression among breast cancer patients undergoing radiotherapy is approximately 80% and 25%, respectively (4, 5). Anxiety and depression not only impair quality of life and immune function, but also increase the risks of breast cancer recurrence and metastasis, potentially shortening survival (6). Therefore, early identification and effective intervention for anxiety and depression are crucial for improving quality of life and enhancing survival in breast cancer patients.

Anxiety and depression in breast cancer patients undergoing radiotherapy are influenced by various factors, including age, education, marital status, employment, religion, income, family history, and surgical history (7, 8). Personality refers to the unique and stable patterns of thinking and behavior that distinguish an individual from others and is widely recognized as a key factor associated with anxiety and depression in psychology (9). Furthermore, personality characteristics are associated with the psychological adjustment in breast cancer patients, which may assist in early identification of emotionally vulnerable individuals and enable targeted clinical interventions and psychological support (10). A cross-sectional study showed that neuroticism was positively correlated with anxiety and depression in patients following breast conserving therapy (11). However, the specific pathways through which personality characteristics are associated with anxiety and depression in breast cancer patients undergoing radiotherapy remain unclear, particularly the potential mediating pathways involved.

Alexithymia refers to difficulty in identifying and describing emotions, a tendency to focus on external details, and limited imaginative capacity (12). Previous research has reported a positive correlation between alexithymia and both anxiety and depression, suggesting that patients with more severe alexithymia may experience more severe symptoms of anxiety and depression (13). A study examined the association between social desirability, alexithymia and neuroticism, as it has been observed that alexithymia can be influenced by higher neuroticism scores (14, 15). A study of 651 participants highlighted the role of alexithymia in understanding personality dysfunction, finding that all dimensions of alexithymia, are associated with various maladaptive personality characteristics (16). Thus, we speculated that alexithymia may play a significant role in the relationship between personality characteristics and anxiety and depression among breast cancer patients undergoing radiotherapy.

From a theoretical perspective, alexithymia may function as an emotional processing bottleneck between stable personality vulnerabilities and state-level emotional symptoms. According to the cognitive-affective processing model of alexithymia, individuals with high alexithymia experience emotional arousal but fail to symbolically process, differentiate, and regulate these sensations into manageable emotional experiences (17). This deficit in symbolic processing may explain why certain personality traits translate into anxiety and depression in some individuals but not others (13, 17). Furthermore, the radiotherapy period represents a unique stress convergence point in the breast cancer trajectory. Unlike the diagnostic phase, which is characterized by uncertainty, or the post-treatment survivorship phase, which involves reintegration, radiotherapy involves the simultaneous experience of acute physical symptoms (fatigue, dermatitis, pain) and persistent existential threat (fear of recurrence) (3). This combination places maximal demands on emotion regulation capacities (18), making alexithymia particularly relevant during this stage.

The purpose of this study was to explore the relationship between personality characteristics and anxiety and depression in breast cancer patients undergoing radiotherapy and to assess the indirect effects of alexithymia in this relationship. The hypotheses were as follows: a)personality characteristics of breast cancer patients undergoing radiotherapy are associated with anxiety and depression; b) Alexithymia shows significant indirect effects in the relationship between personality characteristics and anxiety and depression. These findings may contribute to a deeper understanding of emotional disorders and support the development of more targeted interventions to improve the quality of life and enhance survival in breast cancer patients.

## 2 Methods

### 2.1 Study design

This cross-sectional study was conducted in the Department of Radiation Oncology, West China Hospital, Sichuan University from January 2024 to May 2024. The study was performed in accordance with the ethical standards of the Declaration of Helsinki and was approved by the Ethics Committee on Biomedical Research, West China Hospital of Sichuan University (Approval Number: 20231357). All participants provided written informed consent before enrollment.

### 2.2 Participants and procedure

The participants were breast cancer patients who were currently receiving radiotherapy after surgery, and they were selected using convenient sampling methods. Patients completed the survey during the last 2 weeks of radiotherapy. To ensure adequate statistical power for testing the hypothesized mediation model, a sample-size calculation was performed. Based on a widely accepted heuristic of 10–20 participants per variable for multivariate analysis (19), the intended model comprised 14 variables (4 personality characteristics dimensions, 4 alexithymia dimensions, 2 outcome measures, and 4 demographic/clinical covariates), yielding an initial target of 140–280 participants. After allowing for 10% incomplete data, we planned to recruit 154–308 participants. The inclusion criteria for participants were as follows (1): Female breast cancer patients aged 18 years or older (2); Undergoing adjuvant radiotherapy. Exclusion criteria included (1): A documented history of a formally diagnosed psychiatric disorder, verified through both electronic medical record review and patient self-report during eligibility screening (2); Unable to complete the questionnaire independently.

The data collection process was carried out by researchers who received standardized training to ensure consistency and reliability. The general characteristics included demographic and disease-related characteristics. Demographic characteristics comprised age, education, family income, marital status and the number of children. Disease-related characteristics included family history, tumor location, type of surgery, TNM stage, and molecular subtypes. Before the formal investigation, researchers ensured that participants fully understood the purpose and significance of the study. Participants were then asked to voluntarily sign an informed consent form, confirming their understanding and agreement to participate. Subsequently, participants independently completed the questionnaire, which took approximately 20 to 30 minutes. Researchers provided timely guidance and clarifications on-site as needed. After completion, the questionnaires were promptly collected and carefully reviewed to ensure that any missing data were addressed. Disease-related data were collected directly through the hospital’s internal system, as participants often had difficulty accurately completing this information.

### 2.3 Measures

#### 2.3.1 Anxiety and depression

Anxiety and depression were assessed using the Hospital Anxiety and Depression Scale (HADS) (20). The HADS is a validated screening instrument designed to assess symptom severity and identify probable cases, not to establish clinical diagnoses (21). It consists of 14 items, with 7 items for anxiety and 7 for depression. Each item is rated on a 4-point scale (0–3), with descriptive anchors specific to the item content (e.g., 0 = “not at all” to 3 = “most of the time”), and higher scores reflect more severe symptoms. A score of < 8 indicates no probable anxiety or depression, while a score of ≥ 8 suggests probable anxiety or depression. The HADS has demonstrated good psychometric properties across diverse populations. Bjelland et al. (21) reported in their review that Cronbach’s alpha ranges from 0.68 to 0.93 for the anxiety subscale and from 0.67 to 0.90 for the depression subscale across community and clinical samples. In this study, Cronbach’ s α was 0.77 for anxiety and 0.66 for depression.

#### 2.3.2 Personality characteristics

Personality characteristics were assessed using the Eysenck Personality Questionnaire (EPQ). The Chinese version used in this study was revised and verified by Gong (22). The EPQ consists of four dimensions: Neuroticism (N), Extraversion (E), Psychoticism (P) and Lie (L). The E dimension reflects the introversion-extraversion spectrum, assessing whether individuals tend toward introversion or extroversion. The N dimension evaluates emotional stability. The P dimension gauges the degree of stubbornness or interpersonal detachment. The L dimension is a validity scale measuring social desirability; questionnaires with L scale scores exceeding 70 are considered invalid. The scale comprises 88 items in total (24 for N, 21 for E, 23 for P and 20 for L). Each item is answered “yes” or “no” and scored as 0 or 1. Dimension scores are summed and converted to standard scores based on established norms: scores of 43.3–56.7 indicate intermediate characteristics; scores of 38.5–43.3 or 56.7–61.5 suggest tendency characteristics; and scores below 38.5 or above 61.5 indicate typical characteristics. The EPQ has demonstrated good psychometric properties in Chinese community samples, with reported internal consistency coefficients ranging from 0.68 to 0.81 and test-retest reliabilities ranging from 0.83 to 0.90 (22). In the present study, the Cronbach’ s α values were 0.87 for N, 0.69 for E, 0.74 for P, and 0.70 for L.

#### 2.3.3 Alexithymia

The Toronto Alexithymia Scale (TAS-26) (23, 24) was developed by Taylor to measure the presence and severity of alexithymia. The Chinese version, revised by Yao et al. (25), consists of 26 items with four subscales: Difficulties Identifying Feelings (DIF, 7 items), Difficulties Describing Feelings (DDF, 6 items), Externally Oriented Thinking (EOT, 8 items), and Difficulties Fantasizing (DFAN, 5 items). Each item is rated on a 5-point Likert scale ranging from 1 (‘completely disagree’) to 5 (‘completely agree’), with higher total scores indicating greater alexithymia severity. The Chinese version has demonstrated acceptable psychometric properties in community samples (25). In this study, the Cronbach’s α values were 0.66 for DIF, 0.67 for DDF, 0.65 for EOT, 0.62 for DFAN.

### 2.4 Statistical analyses

Data analysis was performed using SPSS version 27.0. HADS scores were dichotomized (≥ 8) solely for descriptive analyses and were treated as continuous variables in all correlation and mediation analyses. Continuous data are presented as means and standard deviations, while categorical data are presented as frequencies and percentages. The chi-square test was used to assess differences in anxiety and depression across participants’ general characteristics. Pearson’s correlation coefficients were used to analyze the correlations of anxiety and depression with personality characteristics and alexithymia. Mediation analysis was conducted using the PROCESS macro (Model 4) developed by Hayes. Each personality dimension (Neuroticism, Extraversion, and Psychoticism) was entered as the independent variable in separate models, with alexithymia as the mediator, and anxiety and depression as the dependent variables, respectively. Thus, a total of six mediation models were tested (three personality characteristics × two outcomes). Education and molecular subtype were included as covariates: education in all models, and molecular subtype additionally in depression models. All reported coefficients are standardized (β). The bootstrap method with 5,000 resamples was used to estimate the 95% confidence intervals (CIs) for indirect effects. An indirect effect was considered statistically significant if the 95% CI did not include zero. Statistical significance was set at *P* <.05.

## 3 Results

### 3.1 Participants characteristics

A total of 248 eligible patients agreed to participate and completed the questionnaire during the last two weeks of their radiotherapy. Of these, 17 were excluded: 14 due to Lie scale scores > 70 on the EPQ and 3 due to incomplete data, yielding a final analytical sample of 231 participants (valid response rate: 93.1%). The characteristics of the participants are shown in **Table 1**. The majority of participants (46.8%) were aged 50–59 years. Regarding education, 47.6% had completed middle school or less, and 58.9% had a monthly family income of less than 5,000 RMB. Additionally, 93.5% of the participants were married, and 71% had one child. The most common tumor site was the outer quadrant of the left breast. Among the participants, 77.5% underwent radical mastectomy, while 22.5% underwent breast-conserving surgery. A family history of breast cancer was present in 6.5% of participants, and 52.8% were classified as the Luminal B subtype.

**Table 1:** The demographic and disease-related characteristics of the participants.

Characteristics	N (%)
Age(years)	
<40	24 (10.4)
40-49	74 (32.0)
50-59	108 (46.8)
≥60	25 (10.8)
Education	
Middle school and below	110 (47.6)
High school	54 (23.4)
College and above	67 (29.0)
Family income (RMB/month)	
<5000	136 (58.9)
5000-10000	71 (30.7)
>10000	24 (10.4)
Marriage	
Married	216 (93.5)
Unmarried	15 (6.5)
Children	
0	13 (5.6)
1	164 (71.0)
≥2	54 (23.4)
Family history	
No	216 (93.5)
Yes	15 (6.5)
Tumor location	
Left inner quadrant	42 (18.2)
Left outer quadrant	97 (42.0)
Right inner quadrant	25 (10.8)
Right outer quadrant	67 (29.0)
Type of surgery	
Breast-conserving surgery	52 (22.5)
Radical mastectomy	179 (77.5)
T stage	
1	108 (46.8)
2	101 (43.7)
≥3	22 (9.5)
N stage	
0	77 (33.3)
1	106 (45.9)
≥2	48 (20.8)
Molecular subtype	
Luminal A subtype	45 (19.5)
Luminal B subtype	122 (52.8)
HER2-overexpressing subtype	42 (18.2)
Triple-negative subtype	22 (9.5)

### 3.2 Assessment of anxiety, depression, personality characteristics and alexithymia

Descriptive statistics were used to calculate the mean scores and standard deviations for anxiety, depression, personality characteristics, and alexithymia; the results are presented in **Table 2**. The mean score for anxiety was 5.64 (± 4.24), and the mean score for depression was 7.52 (± 4.27). A cutoff score of ≥ 8 was used to identify anxiety or depression. Based on this threshold, 80 participants (34.6%) were classified as having probable anxiety, and 119 participants (51.5%) were classified as having probable depression.

**Table 2:** Scores on anxiety, depression, personality characteristics and alexithymia (n=231).

Variables	Mean ± SD	Min-Max
Anxiety	5.64 ± 4.24	0-21
Depression	7.52 ± 4.27	0-21
Neuroticism dimension	52.19 ± 11.07	25-75
Extraversion dimension	55.89 ± 8.47	35-80
Psychoticism dimension	54.07 ± 12.52	35-100
Total score of alexithymia	74.16 ± 10.38	42-94
Difficulties Identifying Feelings	22.19 ± 6.01	7-35
Difficulties Describing Feelings	18.02 ± 5.66	6-30
Externally Oriented Thinking	18.03 ± 4.31	8-36
Difficulties Fantasizing	15.92 ± 4.44	5-25

SD, Standard Deviation.

### 3.3 Differences in anxiety and depression based on demographic and disease-related characteristics

Differences in anxiety and depression based on demographic and disease-related characteristics are presented in **Table 3**. The results showed that several factors, including age, family income, marital status, number of children, family history, tumor location, type of surgery, T stage, and N stage, were not significantly associated with probable anxiety and depression. However, education level emerged as a significant factor (*P* = .001). Specifically, patients with ≤ middle school or ≥ college education exhibited higher rates of probable anxiety and depression, whereas those with a high school education reported lower levels. Additionally, molecular subtype was significantly associated with depression (*P* = .007), with patients of the luminal A subtype exhibiting a lower incidence of depression compared to those with other subtypes. Based on these findings, education level was included as a covariate in all mediation models, and molecular subtype was additionally controlled for in the depression models.

**Table 3:** Differences in probable anxiety and depression based on demographic and disease-related characteristics (n=231).

Characteristics	Anxiety, n (%)				Depression, n (%)			
	No (n=151)	Yes (n=80)	*χ* ^2^	*P* value	No (n=112)	Yes (n=119)	*χ* ^2^	*P* value
Age(year)								
<40	15 (9.93)	9 (11.25)	4.011	.260	15 (13.39)	9 (7.56)	3.271	.352
40-49	55 (36.42)	19 (23.75)			38 (33.93)	36 (30.25)		
50-59	65 (43.05)	43 (53.75)			49 (43.75)	59 (49.58)		
≥60	16 (10.60)	9 (11.25)			10 (8.93)	15 (12.61)		
Education								
Middle school and below	62 (41.06)	48 (60.00)	13.349	.001	40 (35.71)	70 (58.82)	13.454	.001
High school	46 (30.46)	8 (10.00)			35 (31.25)	19 (15.97)		
College and above	43 (28.48)	24 (30.00)			37 (33.04)	30 (25.21)		
Family income (RMB/month)								
<5000	82 (54.30)	54 (67.50)	4.366	.113	57 (50.89)	79 (66.39)	5.723	.057
5000-10000	50 (33.11)	21 (26.25)			41 (36.61)	30 (25.21)		
>10000	19 (12.58)	5 (6.25)			14 (12.50)	10 (8.40)		
Marriage								
Married	143 (94.70)	73 (91.25)	1.026	.311	106 (94.64)	110 (92.44)	0.042	.497
Single/divorced/widowed	8 (5.30)	7 (8.75)			6 (5.36)	9 (7.56)		
The number of children								
0	9 (5.96)	4 (5.00)	0.605	.739	7 (6.25)	6 (5.04)	1.051	.591
1	109 (72.19)	55 (68.75)			82 (73.21)	82 (68.91)		
≥2	33 (21.85)	21 (26.25)			23 (20.54)	31 (26.05)		
Family history								
No	142 (94.04)	74 (92.50)	0.204	.651	103 (91.96)	113 (94.96)	0.852	.356
Yes	9 (5.96)	6 (7.50)			9 (8.04)	6 (5.04)		
Tumor location								
Left inner quadrant	25 (16.56)	17 (21.25)	1.477	.688	23 (20.54)	19 (15.97)	3.507	.320
Left outer quadrant	62 (41.06)	35 (43.75)			41 (36.61)	56 (47.06)		
Right inner quadrant	17 (11.26)	8 (10.00)			15 (13.39)	10 (8.40)		
Right outer quadrant	47 (31.12)	20 (25.00)			33 (29.46)	34 (28.57)		
Type of surgery								
Breast-conserving surgery	38 (25.17)	14 (17.50)	1.762	.184	29 (25.89)	23 (19.33)	1.426	.232
Radical mastectomy	113 (74.83)	66 (82.50)			83 (74.11)	96 (80.67)		
T stage								
1	71 (47.02)	37 (46.25)	0.036	.982	52 (46.43)	56 (47.06)	0.674	.714
2	66 (43.71)	35 (43.75)			51 (45.54)	50 (42.02)		
≥3	14 (9.27)	8 (10.00)			9 (8.04)	13 (10.92)		
N stage								
0	50 (33.11)	27 (33.75)	5.625	.06	37 (33.04)	40 (33.61)	0.238	.888
1	76 (50.33)	30 (37.50)			53 (47.32)	53 (44.54)		
≥2	25 (16.56)	23 (28.75)			22 (19.64)	26 (21.85)		
Molecular subtype								
Luminal A subtype	31 (20.53)	14 (17.50)	1.313	.726	31 (27.68)	14 (11.76)	11.985	.007
Luminal B subtype	76 (50.33)	46 (57.50)			52 (46.43)	70 (58.82)		
HER2-overexpressing subtype	28 (18.54)	14 (17.50)			16 (14.29)	26 (21.85)		
Triple-negative subtype	16 (10.60)	6 (7.50)			13 (11.61)	9 (7.56)		

### 3.4 Correlation analysis of anxiety, depression, personality characteristics and alexithymia

The correlation analysis (**Table 4**) identified significant associations between anxiety, depression, personality characteristics, and alexithymia. Neuroticism and psychoticism were positively correlated with both anxiety (r = 0.471, *P* <.001; r = 0.486, *P* <.001) and depression (r = 0.246, *P* <.05; r = 0.571, *P* <.001), while extraversion showed a negative correlation with depression (r = -0.208, *P* <.01). Higher levels of alexithymia were associated with increased anxiety (r = 0.294, *P* <.001) and depression (r = 0.407, *P* <.001). Furthermore, neuroticism (r = 0.162, *P* <.05) and psychoticism (r = 0.359, *P* <.001) were positively correlated with alexithymia, whereas extraversion was negatively correlated with alexithymia (r = -0.178, *P* <.01).

**Table 4:** Correlation analysis of anxiety, depression, personality characteristics and alexithymia.

Variables	Anxiety	Depression	Neuroticism	Extraversion	Psychoticism	Alexithymia
Anxiety	1					
Depression	0.587^c^	1				
Neuroticism	0.471^c^	0.246^a^	1			
Extraversion	-0.053	-0.208^b^	-0.003	1		
Psychoticism	0.486^c^	0.571^c^	0.123	-0.019	1	
Alexithymia	0.294^c^	0.407^c^	0.162^a^	-0.178^b^	0.359^c^	1

^a^ *P* <.05.

^b^ *P* <.01.

^c^ *P* <.001.

### 3.5 Mediating effect of alexithymia on anxiety, depression and personality characteristics

**Figure 1:**
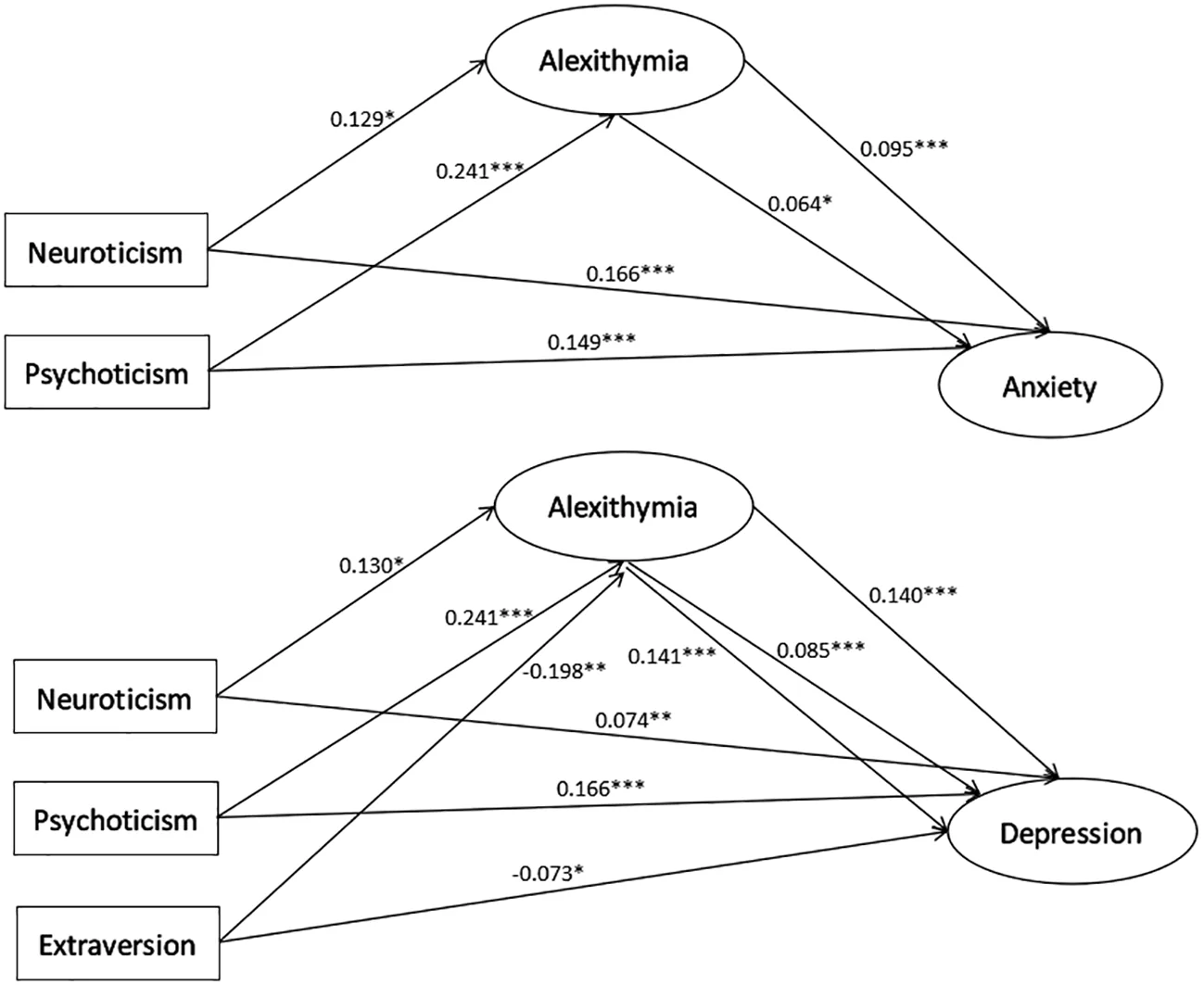
Mediation models of alexithymia in the relationships between personality characteristics and anxiety (top) and depression (bottom) in breast cancer patients undergoing radiotherapy. The beta values on the paths represent standardized regression coefficients. *P <.05, **P <.01, ***P <.001.

Personality characteristics were set as the independent variable, anxiety and depression as the dependent variables, and alexithymia as the mediating variable to analyze their mediating effects. Given the significant correlations between education and both anxiety and depression, as well as between molecular subtype and depression, in the demographic analyses, we controlled for education as a covariate in all mediation models and additionally included molecular subtype as a covariate in the depression models. **Figure 1** illustrates the standardized path coefficients for two separate mediation models. For anxiety, neuroticism and psychoticism both showed significant direct effects (β= 0.166 and β= 0.149, respectively, both *P* <.001) and significant indirect effects via alexithymia (β= 0.012 and β= 0.015, respectively, 95% CIs excluded 0). Notably, psychoticism demonstrated a stronger association with alexithymia (β= 0.241) than neuroticism (β= 0.129). For depression, similar patterns were observed, with extraversion additionally showing a negative indirect effect through lower alexithymia (β= −0.027, 95% CI excluded 0). **Table 5** presents the complete mediation analysis results, confirming that all indirect effects of alexithymia were statistically significant.

**Table 5:** The mediation analysis of alexithymia as a mediator between personality characteristics and anxiety and depression.

Path	Effect	Effect value	SE	LLCI	ULCI	Effect size
Neuroticism→Alexithymia→Anxiety	Total effects	0.178	0.022	0.135	0.223	
	Direct effects	0.166	0.022	0.123	0.209	93%
	Indirect effects	0.012	0.006	0.002	0.026	7%
Psychoticism→Alexithymia→Anxiety	Total effects	0.164	0.020	0.125	0.203	
	Direct effects	0.149	0.021	0.108	0.189	91%
	Indirect effects	0.015	0.008	0.002	0.032	9%
Neuroticism→Alexithymia→Depression	Total effects	0.092	0.024	0.045	0.140	
	Direct effects	0.074	0.023	0.029	0.119	80%
	Indirect effects	0.018	0.009	0.003	0.038	20%
Psychoticism→Alexithymia→Depression	Total effects	0.186	0.019	0.149	0.223	
	Direct effects	0.166	0.019	0.128	0.203	89%
	Indirect effects	0.020	0.008	0.007	0.038	11%
Extraversion→Alexithymia→Depression	Total effects	-0.101	0.032	-0.163	-0.039	
	Direct effects	-0.073	0.031	-0.133	-0.013	72%
	Indirect effects	-0.028	0.012	-0.053	-0.008	28%

SE, Standard Error; LLCI, Lower Level of Confidence Interval; ULCI, Upper Level of Confidence Interval.

## 4 Discussion

In this study, the rates of probable anxiety and depression among breast cancer patients undergoing radiotherapy after surgery were 34.6% and 51.5%, respectively, which are higher than those reported in a previous systematic review of breast cancer patients in southwest China who were not undergoing radiotherapy, where the prevalence rates were 12% for anxiety and 19% for depression (26). These discrepancies may be related to the additional physical burden and psychological stress associated with radiotherapy. Furthermore, differences in the assessment tools used may also contribute to the observed variations in results.

We also explored the differences in anxiety and depression based on various demographic and disease-related characteristics, and found that the anxiety and depression levels varied among breast cancer patients undergoing radiotherapy after surgery, depending on their education level. Patients with education levels of ≤ middle school or ≥ college exhibited higher rates of probable anxiety and depression. This finding may be explained by differing psychological processes. Patients with lower education may experience distress due to limited health literacy and reduced access to coping resources (27). Conversely, those with higher education may be more vulnerable to information overload and heightened existential concerns when facing a life-threatening illness (28). However, these interpretations remain speculative and warrant further investigation. In addition, there are differences in the degree of depression among breast cancer patients with different molecular subtypes. Patients with luminal B, HER2-overexpressing, and triple-negative, compared to those with the luminal A subtype, tend to have poorer prognoses and may be more prone to depression (29). Consequently, these patients may require more psychological support and intervention to improve their mental health status. This highlights the complex interplay between demographic, clinical, and molecular factors in shaping the psychological well-being of breast cancer patients undergoing radiotherapy.

The study found that anxiety was significantly positively correlated with neuroticism and psychoticism in breast cancer patients undergoing radiotherapy after surgery. This finding is consistent with previous research (9) and suggests that these personality characteristics may jointly form the psychological substrate of anxiety (30). Individuals with higher levels of neuroticism are often characterized by emotional instability and a tendency toward negative emotional experiences (31). Concurrently, psychoticism is associated with antisocial behavior and impulsivity, both of which may increase the likelihood of anxiety (32). A significant positive correlation was also found between depression and both neuroticism and psychoticism. Neuroticism is a well-established risk factor for depression; individuals with high neuroticism are more likely to experience persistent negative emotions and pessimistic thinking. Individuals with high psychoticism may be more prone to depression due to their traits of indifference and alienation (33). Notably, a significant negative correlation was observed between depression and extraversion. This suggests that extraversion may be associated with lower depression levels, potentially related to its occurrence. In contrast, introverted individuals may be more introspective and solitary, which may be associated with limited access to social support and potentially higher depression risk (34).

The study suggests that alexithymia shows significant indirect effects between the personality characteristics of neuroticism and psychoticism and the emotions of anxiety and depression in breast cancer patients undergoing radiotherapy after surgery. This statistical mediation pattern could be interpreted within the framework of Gross’s process model of emotion regulation (18). According to this framework, effective emotion regulation requires sequential stages from situation selection to response modulation, with cognitive change (particularly cognitive reappraisal) being critical for modulating emotional outcomes. Alexithymia may specifically affect the transition from emotional arousal to cognitive change—patients may perceive treatment-related distress but may have difficulty differentiating, labeling and symbolically processing these sensations into manageable emotional experiences (17). Consequently, individuals high in neuroticism or psychoticism may experience intensified emotional arousal due to radiotherapy stressors. However, when co-occurring alexithymia impedes the implementation of reappraisal, this arousal could accumulate as undifferentiated negative affect, potentially contributing to anxiety and depression (35).

Notably, psychoticism demonstrated a stronger association with alexithymia (β= 0.241) than neuroticism (β=0.129). This differential pattern may reflect distinct pathways linking these traits to emotional processing. Psychoticism’s core features—interpersonal detachment and reality distortion—may fundamentally affect the symbolic processing required for emotion regulation, which could make these patients particularly susceptible to alexithymia-mediated pathways associated with anxiety and depression (17). In contrast, neuroticism primarily reflects hyper-reactivity to negative stimuli; these patients may experience intense emotions but may retain some capacity to identify them, which could explain their relatively weaker reliance on alexithymia as a mediating pathway. The protective role of extraversion operates through lower alexithymia (β= -0.198), consistent with the social sharing of emotion model (36): extraverted individuals’ tendency toward verbal emotional expression may facilitate the labeling and processing of emotional experiences, thereby bypassing the alexithymic bottleneck that otherwise contributes to depression. These differential pathways illustrate the diverse processes through which personality characteristics may be associated with psychological adaptation via emotional processing capacities, providing a theoretical foundation for future longitudinal research and the development of targeted prevention strategies.

The present findings are compatible with broader theoretical frameworks emphasizing emotion regulation in oncology populations. Bacık Yaman et al. (37) reported that perceived stress and emotion regulation skills are associated with attachment styles after cancer diagnosis, highlighting the importance of emotion regulation in psychological adjustment. Similarly, Bacık Yaman et al. (38) found associations among death anxiety, anger, anxiety, and depressive symptoms in elderly cancer patients. Evidence from chronic conditions beyond oncology also suggests that impaired emotional awareness may operate as a transdiagnostic correlate: Alkaş et al. (39) observed elevated alexithymia levels in Restless Legs Syndrome patients, with alexithymia positively associated with disease severity. While these findings suggest that alexithymia may be a shared psychological marker across chronic physical illnesses, this hypothesis requires longitudinal validation. The present findings are consistent with, but do not demonstrate, these broader frameworks.

This study has several limitations. First, the cross-sectional design limits the ability to infer causality. Future studies should employ longitudinal designs to better understand changes in personality characteristics, alexithymia, anxiety and depression over time. Second, this study did not explore the relationship between anxiety, depression, personality characteristics and alexithymia at different time points during radiotherapy. Stratified analyses based on treatment timeline could reveal the dynamic changes and trajectories of patients’ psychological states throughout the course of radiotherapy. Third, all data were collected using self-report questionnaires, which may be subject to recall bias, social desirability bias, and common method variance. Future research could incorporate clinician-administered assessments or ecological momentary assessments to minimize these biases. Furthermore, some subscales, particularly HADS-Depression and TAS-DFAN, showed marginal internal consistency (α < 0.70), which may have attenuated the observed associations and should be considered when interpreting the results. Fourth, the sample-size calculation was based on a general heuristic rather than a method specifically designed for mediation models. Although our sample of 231 participants is comparable to those in similar studies, future research with *a priori* power analyses for indirect effects would further strengthen the evidence. Finally, future research may also consider biomarkers and neuroimaging data to further explore the biological mechanisms underlying these psychological factors. Additionally, we did not collect data on systemic treatments (e.g., chemotherapy, endocrine therapy), which may influence psychological outcomes and warrant future investigation.

Clinically, our findings suggest that screening for alexithymia in radiotherapy units could identify patients at risk for emotional distress. Brief emotion regulation training or psychoeducation focused on identifying and verbalizing emotions may be particularly beneficial for patients with high neuroticism or psychoticism, potentially mitigating psychological symptoms during radiotherapy.

## 5 Conclusions

This cross-sectional study found that neuroticism and psychoticism were positively associated with anxiety and depression in breast cancer patients undergoing postoperative radiotherapy, while extraversion was negatively associated with depression. Alexithymia showed significant indirect associations between selected personality characteristics and psychological distress. These findings suggest that alexithymia may be a clinically relevant psychological marker in this population; however, longitudinal studies are needed to confirm temporal and causal pathways.

## Data Availability

The raw data supporting the conclusions of this article will be made available by the authors, without undue reservation.
